# Comparison of laparoscopic hepatectomy and percutaneous radiofrequency ablation for the treatment of small hepatocellular carcinoma: a meta-analysis

**DOI:** 10.1186/s12893-024-02376-5

**Published:** 2024-03-05

**Authors:** Fei Liu, Ling Tan, Lan Luo, Jun-jiang Pan

**Affiliations:** 1 Department of General Surgery, Second People’s Hospital of Yibin City, Yibin, 644000 Sichuan China; 2 Department of Urology, People’s Hospital Affiliated to Chongqing Three Gorges Medical College, Chongqing, 404041 China

**Keywords:** Laparoscopic hepatectomy, Percutaneous radiofrequency ablation, Hepatocellular carcinoma, Meta-analysis

## Abstract

**Aim:**

The purpose of this study was to compare the long-term outcomes of laparoscopic hepatectomy (LH) and percutaneous radiofrequency ablation (PRFA) for the treatment of small hepatocellular carcinoma.

**Methods:**

We systematically searched PubMed, Embase, Web of Science, and Medline from January 2000 to May 2022 for literature comparing the efficacy of LH and PRFA in the treatment of small hepatocellular carcinoma (largest tumour diameter ≤ 3 cm, number of intrahepatic tumours ≤3, or diameter of a single intrahepatic lesion ≤5 cm.

). We assessed overall survival (OS), recurrence-free survival (RFS), local recurrence and complication rates.

**Results:**

A total of 1886 patients with small HCC were included in the 8 studies included in this study, of which 839 underwent LH and 1047 underwent PRAF. The results of the meta-analysis showed that the two groups had the same 3-year (HR: 0.99, 95% CI: 0.67 to 1.47) and 5-year (HR: 1.30, 95% CI: 0.90 to 1.87) OS rates, and the LH group had better 3-year (HR: 0.58, 95% CI: 0.49 to 0.68) and 5-year (HR: 0.56, 95% CI: 0.37 to 0.85) RFS rates. The LH group had a lower local recurrence rate (OR: 0.19, 95% CI: 0.12 to 0.32), but the PRFA group had a lower complication rate (OR: 2.49, 95% CI: 1.76 to 3.54).

**Conclusion:**

There was no difference in OS between LH and PRFA in the treatment of small HCC. LH had a higher RFS rate and a lower local recurrence rate, but PRFA had a lower complication rate. In general, the long-term efficacy of LH in the treatment of small HCC is better than that of PRFA. Considering the advantages of less trauma and a low complication rate of PRFA, a large number of RCT studies are needed for further verification in the future.

**Supplementary Information:**

The online version contains supplementary material available at 10.1186/s12893-024-02376-5.

## Introduction

Hepatocellular carcinoma (HCC) is the sixth most common tumour and the fourth leading cause of cancer-related death in the world, seriously affecting human life and health [1]. With the improvement of imaging diagnosis-related technologies and the popularization of tumour screening procedures, an increasing number of early-stage liver cancer cases are being discovered and have the opportunity to receive treatment [2, 3]. Although liver transplantation (LT) is considered to be the best treatment for small hepatocellular carcinoma [4], the application of LT is limited due to the shortage of donors, the high cost, technical difficulty, and many complications [5].

Hepatectomy is still the preferred treatment for hepatocellular carcinoma [6], but it remains limited by the patient’s liver function and has a high incidence of complications [7, 8]. Radiofrequency ablation has the characteristics of less impact on liver function, less trauma, and repeatability. It can achieve curative effects similar to those of surgical resection in some early-stage liver cancer patients, and it has become one of the first-choice treatment methods for early-stage liver cancer [9, 10]. In the Barcelona Clinic Liver Cancer (BCLC) staging system, both radiofrequency ablation and hepatectomy are recommended for the treatment of early-stage HCC [11].

In recent years, with the improvement of laparoscopic technology and the update of auxiliary equipment, laparoscopic liver resection has achieved rapid development in liver surgery. Similar to RFA, it has the advantages of less trauma, faster recovery, and shorter hospital stays, so it is widely used in the early stage. However, studies have shown that surgical resection is superior to radiofrequency ablation in long-term outcomes in the treatment of small hepatocellular carcinoma [12–15]. There is no conclusion as to whether complete laparoscopic hepatectomy or percutaneous radiofrequency ablation is better in the long-term treatment of small hepatocellular carcinoma, and the choice of the two minimally invasive methods in the treatment of small hepatocellular carcinoma is still controversial. Therefore, we conducted this meta-analysis to evaluate the long-term outcomes of these two minimally invasive modalities in the treatment of small HCC and to provide a reference for clinical treatment decisions.

## Materials and methods

### Search strategy and inclusion criteria

For this meta-analysis, we adhered to the Meta-analysis of Observational Studies guidelines [16] and the Preferred Reporting Items for Systematic Reviews and Meta-Analyses statement [17]. A systematic search was performed based on the PubMed, Embase, Web of Science, and Medline databases from January 1, 2000, to May 31, 2023. We used “hepatocellular carcinoma”, “laparoscopic hepatectomy”, “percutaneous radiofrequency ablation”, “prognosis” and corresponding free words to search the literature in the above databases. Study inclusion and exclusion criteria: 1. Inclusion criteria: 1). Studies on LH and PRFA in the treatment of small liver cancer were compared without limitation; 2). The criteria for tumor size were: maximum tumor diameter ≤ 3 cm, number of intrahepatic tumors ≤3, or diameter of single intrahepatic lesion ≤5 cm; 3). At least one usable data item has been provided; 2. Exclusion criteria: 1). Studies on other surgical methods (such as robotic surgery) were combined; 2). The tumor size does not meet the criteria for small liver cancer or there is no clear study of tumor size; 3). Studies that do not provide usable data.

First, all the identified titles and abstracts were examined by two independent reviewers (Tan L and Liu F). Next, the same two reviewers independently examined the full texts of potentially relevant articles. In the event of a disagreement, a third reviewer (Luo L) was consulted, and the relevant articles were discussed until a consensus was reached.

### Definition of small hepatocellular carcinoma

In this study, small hepatocellular carcinoma was defined as Barcelona Clinic Liver Cancer (BCLC) [18] stage 0 or A, largest tumour diameter ≤ 3 cm, number of intrahepatic tumours ≤3, or diameter of a single intrahepatic lesion ≤5 cm.

### Data extraction and quality assessment

Basic information, such as first author, year of publication, country, number of patients, age, years of follow-up, and type of outcome, were extracted from all included publications. The primary outcome was the prognostic difference in overall survival (OS), recurrence-free survival (RFS), local recurrence, and complications in patients with small HCC treated with LH and PRFA. Therefore, if available, the following data were extracted: hazard ratios (HRs), 95% confidence intervals (CIs) and *P* values of OS and RFS. When the literature did not report HRs, only OS and RFS, K-M curves and Engauge Digitizer (version 10.8) were used to determine the survival rate of the corresponding time points on the curve, followed by the HR calculation table [19]. All data were extracted independently by two authors (Tan L and Liu F) and compared for consistency.

The quality of the included studies was assessed using the Newcastle–Ottawa Scale (NOS), with a maximum of 9 points per study. Publication bias was assessed by visual inspection of the symmetry of the funnel plot. We considered that the heterogeneity in the 5-year RFS was derived from a non-propensity-matched analysis. We performed subgroup analyses of 5-year RFS based on propensity-matched analysis.

### Statistical analysis

We used the R (version 4.1.0) Meta package for meta-analysis. Binary outcome data are reported as HRs with 95% CIs using the Mantel–Haenszel method. Heterogeneity was assessed using the I^2^ statistic, and values above 50% were considered to be considerably heterogeneous. The prior decision to use a random effects model was to account for the considerable heterogeneity assumed between studies.

## Results

**Fig. 1:**
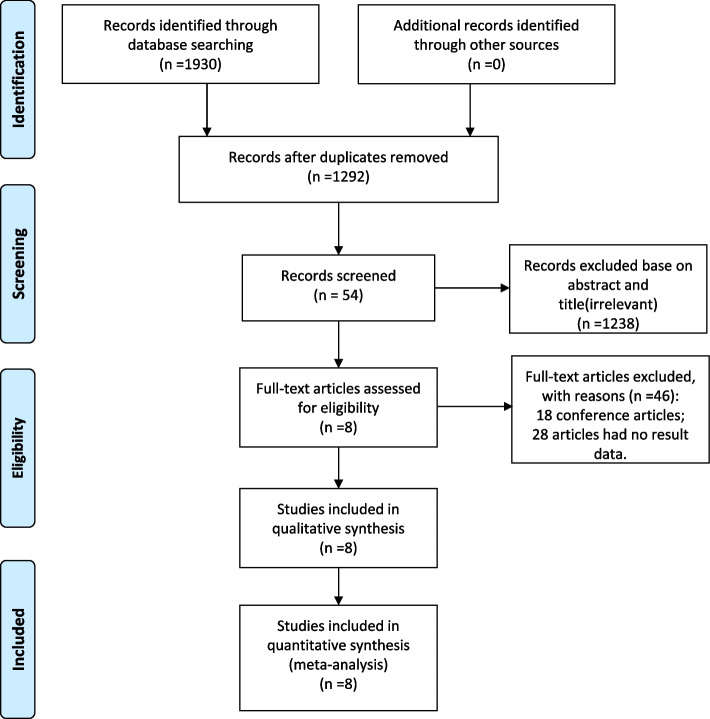
Flowchart of search strategy and study selection. LH and 1047 underwent PRAF. Eight studies had NOS scores ranging from 6 to 8 (Fig. 2). The quality of the included literature was considered qualified

We obtained 1930 publications from databases including PubMed, Medline, Embase, and Web of Science. After removing duplicates, there were 1292 publications. 1238 publications were excluded after reviewing titles and abstracts. The continuing review law excluded 18 conference articles and 28 articles with no relevant results. Finally, a total of 8 publications were eligible for inclusion (Fig. 1). Table 1 shows the basic characteristics of the publications. A total of 1886 patients with small HCC were included in the 8 studies [20–27] included in this study, of which 839 underwent.

**Fig. 2:**
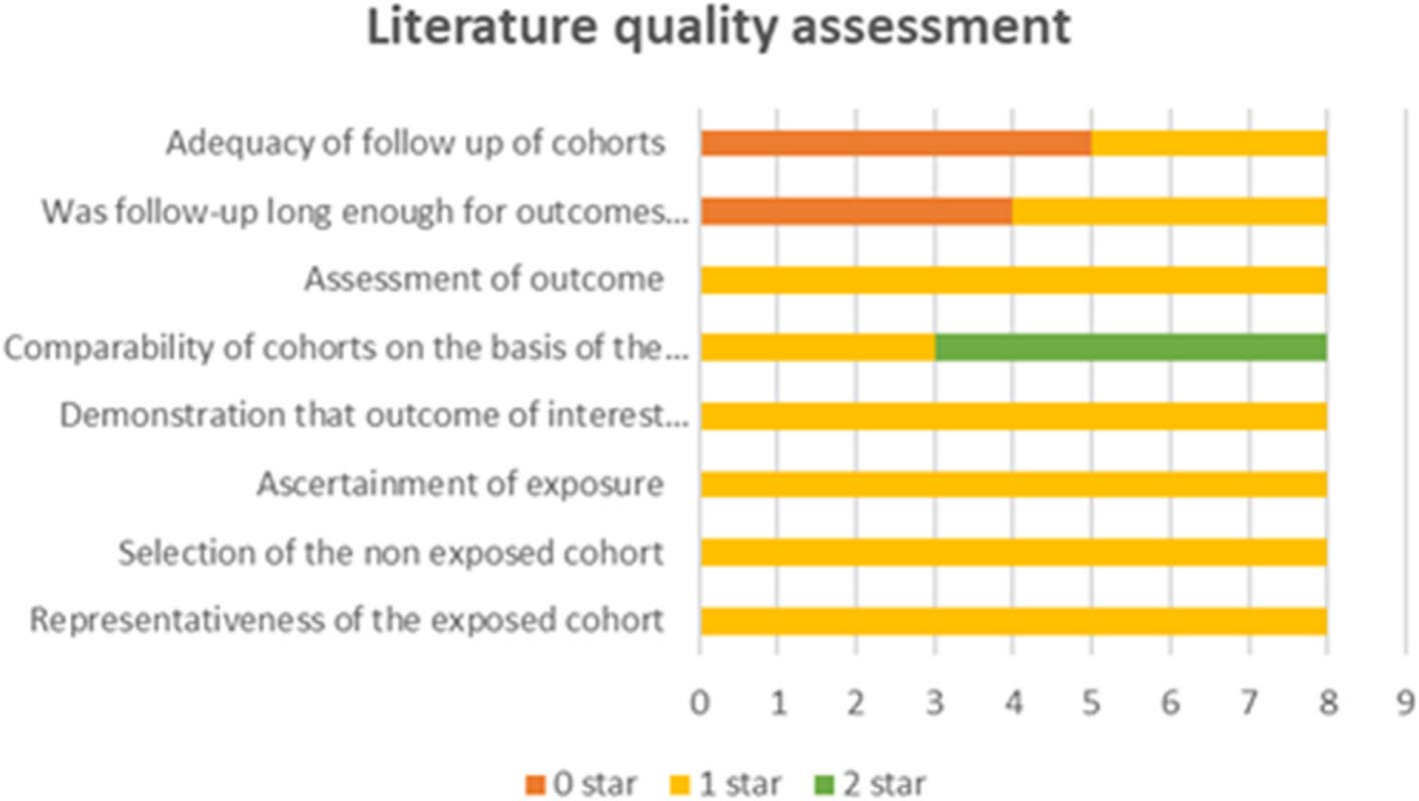
Literature quality assessment based on the Newcastle-Ottawa Scale

**Table 1:** Characteristics of studies included in meta-analysis

Author and year	Journal	Country	Number of patient	Age (years)	Follow-up (month)	Type of study	Outcome
Kai-Chi Cheng 202 2[26]	Transl Cancer Res	China	130	63.60 ± 9.86 vs 65.48 ± 11.73	34(1–175)	RS	OS、RFS
S. Di Sandro 201 9[25]	Eur J Surg Oncol	Italy	182	65(62–72) vs 65(56–76)	33(17–56)	RS	OS、RFS
Satoshi Ogiso 202 1[24]	HPB	Japan	221	69(46–88) vs 73(47–87)	66(1–153) vs 57(1–130)	RS	OS、RFS
Yan-Hua Zhang 202 0[22]	World J Clin Cases	China	175	63.5 ± 7.6 vs 62.8 ± 8.5	24 ± 6	RS	OS、RFS
Dong Ho Lee 202 1[20]	Liver Cancer	Korea	566	57.5 ± 9.3 vs 60.8 ± 9.6	30.0 ± 12.5	RS	RFS
Chong LAI 201 6[21]	J Zhejiang Univ-Sci B	China	94	56.5 ± 12.6 vs 62.8 ± 11.3	36	RS	OS
Yang-xun Pan 202 0[23]	Eur J Surg Oncol	China	477	51 (44–60) vs 57 (46–65)	26.22(1.30–44.77) vs 24.20(0.97–44.73)	RS	OS、RFS
Juxian Song 201 5[27]	Surg Endosc	China	156	48 (44–57) vs 48 (43–58)	31.2(21.1–49.5)	RS	OS、RFS

*RS* Retrospective study: *OS* Overall survival: *RFS* Recurrence-free survival.

### 3-year OS

**Fig. 3:**
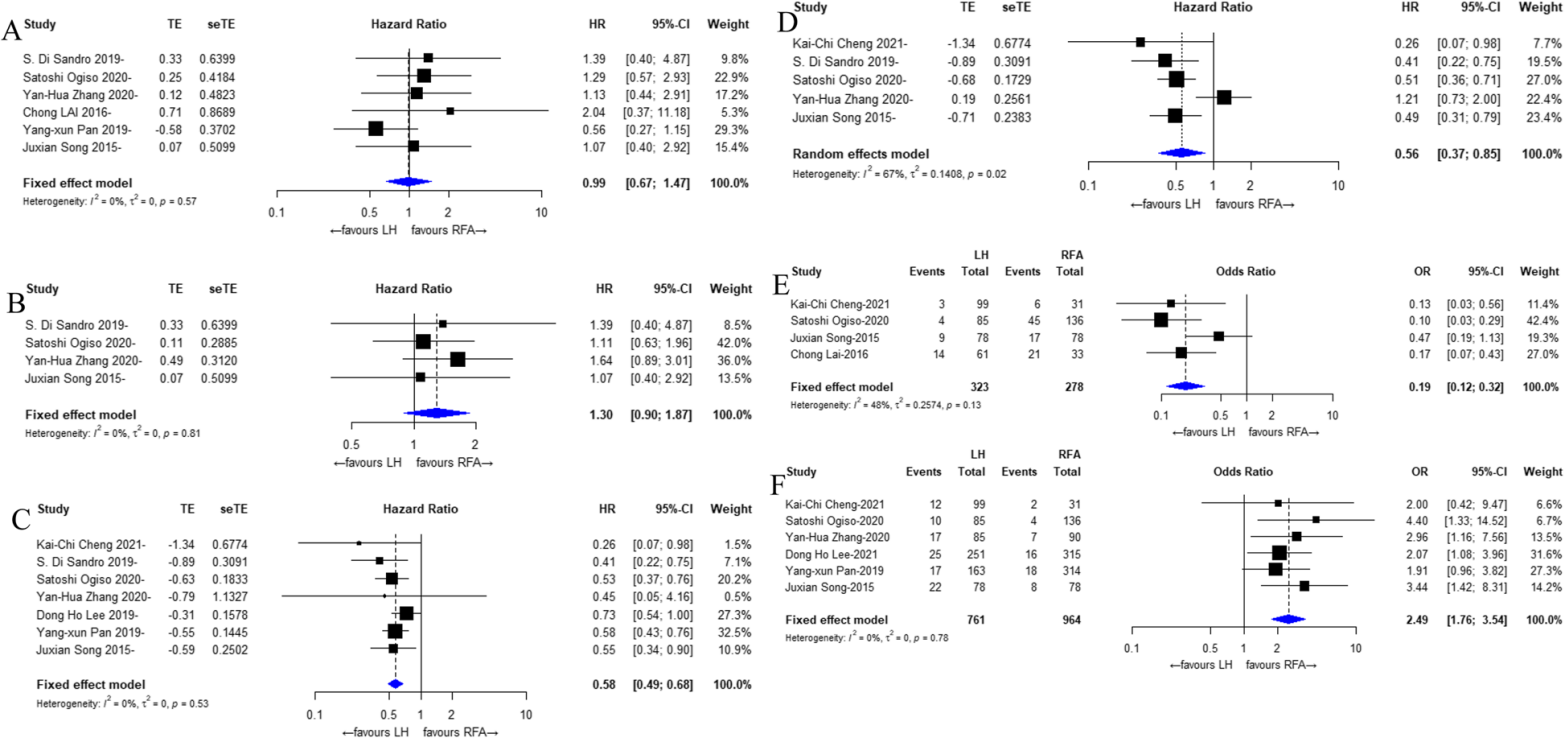
Forest plots for the meta-analyses. **A** 3-year OS; **B** 5-year OS; **C** 3-year RFS; **D **5-year RFS; **E** Local recurrence; **F** Complication

Six [21–25, 27] of the eight included studies reported the 3-year OS results of LH vs. PRFA treatment for small hepatocellular carcinoma, and the overall results showed that there was no significant difference in the 3-year OS between the two treatment strategies of LH and PRFA (HR: 0.99, 95% CI: 0.67 to 1.47, I^2^ = 0%, *P* = 0.57), as shown in Fig. 3A.

### 5-year OS

Four [22, 24, 25, 27] of the eight included studies reported the 5-year OS results of LH vs. PRFA treatment for small hepatocellular carcinoma, and the overall results showed that there was no significant difference in the 5-year OS between the two treatment strategies of LH and PRFA (HR: 1.30, 95% CI: 0.90 to 1.87, I^2^ = 0%, *P* = 0.81), as shown in Fig. 3B.

### 3-year RFS

Seven [20, 22–27] of the eight included studies reported the 3-year RFS results of LH vs. PRFA treatment for small hepatocellular carcinoma. Overall, the results showed that the 3-year RFS was significantly better for small HCC patients treated with LH (HR: 0.58, 95% CI: 0.49 to 0.68, I^2^ = 0%, *P* = 0.53), as shown in Fig. 3C.

### 5-year RFS

Five [22, 24–27] of the eight included studies reported the 5-year RFS results of LH vs. PRFA treatment for small hepatocellular carcinoma. Overall, the results showed that the 5-year RFS was significantly better for small HCC patients receiving LH (HR: 0.56, 95% CI: 0.37 to 0.85, I^2^ = 67%, *P* = 0.02), as shown in Fig. 3D. There was heterogeneity, so we used a random effects model.

### Local recurrence

Four [21, 24, 26, 27] of the eight included studies reported local recurrence outcomes for small hepatocellular carcinoma treated with LH vs. PRFA. The overall results showed that the local recurrence of small HCC treated with LH was significantly less than that of patients treated with PRFA (OR: 0.19, 95% CI: 0.12 to 0.32, I^2^ = 48%, *P* = 0.12), as shown in Fig. 3E.

### Complications

Six [20, 22–24, 26, 27] of the eight included studies reported complication outcomes of LH vs. PRFA treatment for small hepatocellular carcinoma. The overall results showed that LH treatment for small HCC had significantly more complications than PRFA treatment (OR: 2.49, 95% CI: 1.76 to 3.54, I^2^ = 0%, *P* = 0.78), as shown in Fig. 3F.

### Sensitivity analysis

**Fig. 4:**
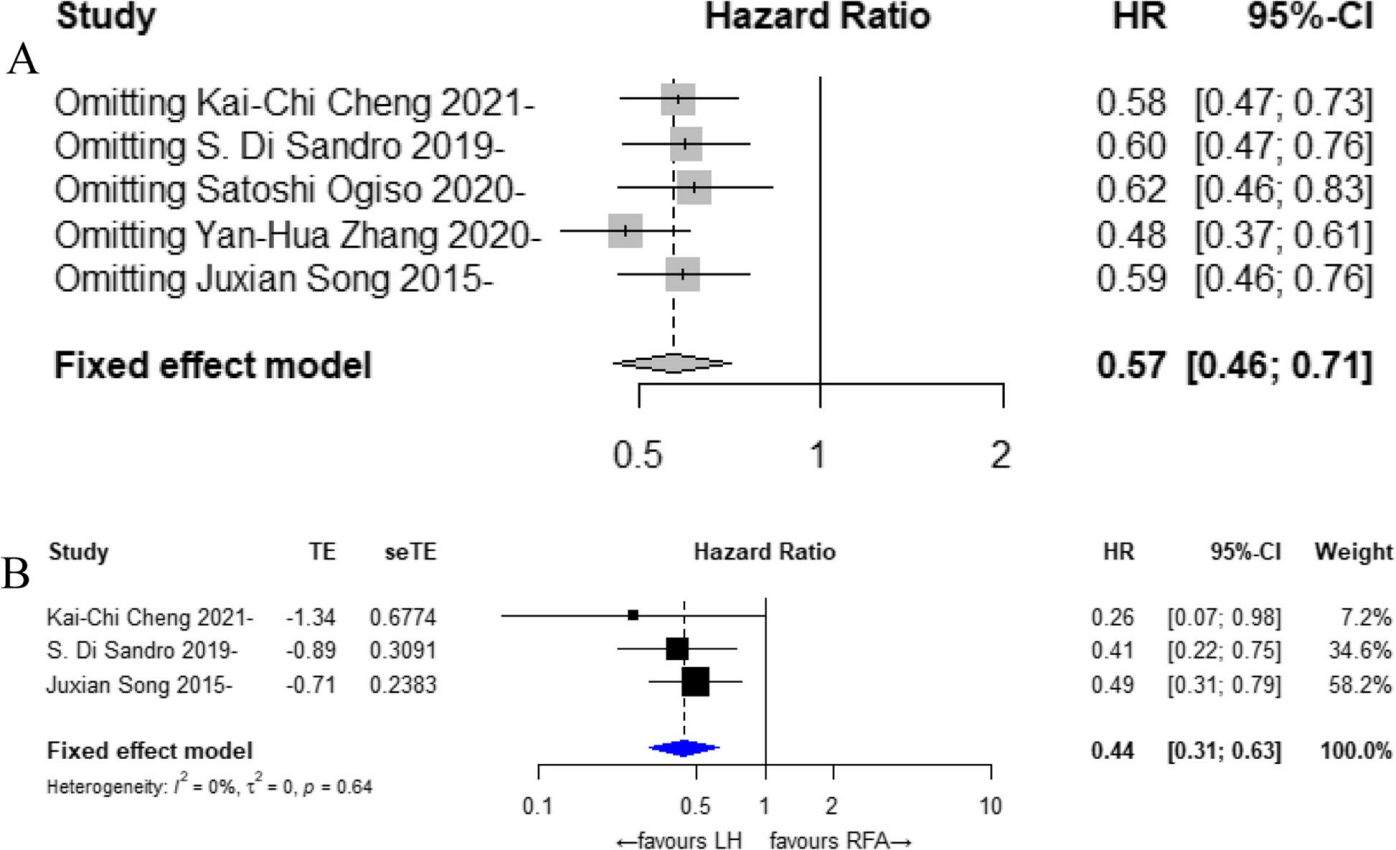
Sensitivity analysis results and subgroup analysis results. **A** Sensitivity analysis; **B** 5-year RFA for subgroup analysis

In our results, there was some heterogeneity in the 5-year RFS, and a sensitivity analysis was performed, as shown in Fig. 4A. We considered that the heterogeneity might arise from the non-propensity-matched analysis, and we performed a subgroup analysis of the propensity-matched analysis. The results showed that 3 [25–27] of the 8 included studies reported the 5-year RFS results of LH vs. PRFA treatment for small hepatocellular carcinoma. The overall results showed that the 5-year RFS of small HCC treated with LH was significantly better than that of small HCC treated with PRFA (HR: 0.44, 95% CI: 0.31 to 0.63, I^2^ = 0%, *P* = 0.64), as shown in Fig. 4B.

### Publication bias

Publication bias was assessed by visual examination of the symmetry of the funnel plot, which showed no publication bias (Supplement Fig. 1).

## Discussion

Surgical resection, liver transplantation, and ablation are methods with high complete remission rates for liver cancer and have curative potential [4]. Liver transplantation is one of the curative treatments for liver cancer, especially for patients with small liver cancer who have decompensated liver function and are not suitable for surgical resection or ablation therapy [28]. However, factors such as a shortage of donors, difficult surgery, and tumour progression during the waiting period greatly limit the widespread implementation of liver transplantation [4, 29]. Therefore, laparoscopic hepatectomy and radiofrequency ablation are widely used in the minimally invasive treatment of small hepatocellular carcinoma, but the choice of these two surgical methods is still controversial [7].

This meta-analysis showed that in terms of survival, the 3-year and 5-year OS rates were comparable between the LH and PRFA groups. Our results are different from those of previous studies. The meta-analyses of Si [15] and Li [30] showed that the 3-year and 5-year OS rates of the minimally invasive liver resection group were better than those of the RFA group. The difference in long-term survival from previous studies may be because the studies of Si and Li did not strictly limit the surgical methods (the previous studies included robotic-assisted liver resection, laparoscopic radiofrequency ablation, and open ablation procedures). Although studies have confirmed that these methods have similar safety and efficacy in the treatment of tumours, there is no final conclusion in terms of the antitumor results [31, 32]. Therefore, the results of previous studies need to be interpreted with caution, and since robotic liver resection has not been widely used, we limited the surgical methods to simple LH and PRFA, two commonly used minimally invasive treatments. There was no difference in long-term survival between the two modalities. This may be because the higher recurrence rate in the locoregional region does not adversely affect overall survival, as recurrence can be further treated with reablation or resection [33, 34].

The RFS results showed that the 3-year and 5-year RFS rates of the LH group were better than those of the PRFA group, and the LH group also had a lower local recurrence rate. However, in terms of the complication rate, the PRFA group had a lower complication rate, which was basically consistent with the results of previous studies. PRFA may be limited by tumour size, location, and adjacent structures, making ablation incomplete and thus more prone to recurrence [15, 35]. Hepatectomy can completely remove the tumour and potential microscopic lesions [36], which may be the reason for the lower recurrence rate in the LH group. The high recurrence rate of PRFA may be due to the heat sink effect [37]. PRFA has mild injury and less bleeding and often does not require general anaesthesia. Patients can eat and move around earlier and have a shorter hospital stay. Therefore, this is a good explanation for why the PRFA group had a lower complication rate. Considering that elderly patients have relatively poor tolerance to surgical trauma and general anesthesia due to reduced functional reserve of multiple organs, and slow recovery after surgery [38], PRFA may be the best surgical method for elderly patients. At the same time, studies have shown that laparoscopic radiofrequency ablation is superior to PRFA [15], which may further broaden the indications of radiofrequency ablation. With the advancement of ablation technology and the improvement of ablation methods and ablation equipment, radiofrequency ablation may become the best treatment method for early small HCC in the future.

We conducted a subgroup analysis of propensity scores, and the results of the subgroup analysis were the same as our original conclusions, which further verified the stability of the results. However, the conclusions of our subgroup analysis need further validation due to the lack of literature included for some indicators.

We believe that this meta-analysis has the following advantages. (1) This is the first meta-analysis comparing percutaneous radiofrequency ablation and laparoscopic liver resection, two commonly used minimally invasive modalities for the treatment of small hepatocellular carcinoma, and the heterogeneity was low for all our results. (2) We performed subgroup analyses to reduce bias due to patient characteristics and tumour factors. (3) The HR (hazard ratio) is the most appropriate parameter to measure time-dependent outcomes [14], so we extracted the HR instead of the OR to calculate OS and RFS.

However, our results also need to be interpreted with caution. First, we have not collected relevant RCT studies, which undoubtedly reduces the strength of the evidence. Second, because most liver cancer patients often have different degrees of liver cirrhosis [39], and the incidence of perioperative complications of liver cirrhosis is significantly increased [40–42], and only some patients with small liver cancer can be treated with surgery [43] . Although we conducted a subgroup analysis of propensity score, due to the lack of included literature, our study may still have larger selection bias.

## Conclusion

This comprehensive literature analysis shows that the long-term efficacy of LH in the treatment of small hepatocellular carcinoma is better than that of PRFA, but considering the advantages of less trauma and a lower complication rate of PRFA, large RCT studies are needed for further verification in the future.

### Supplementary Information

**Supplementary Material 1.**

## Data Availability

All the data are available without restriction. Researchers can obtain data by contacting the corresponding author.

## References

[1] Villanueva A (2019). Hepatocellular Carcinoma. N Engl J Med..

[2] Nowicki TK, Markiet K, Szurowska E (2017). Diagnostic imaging of hepatocellular carcinoma - a pictorial essay. Curr med imag rev..

[3] Liu LF, Ding ZL, Zhong JH, Li HX, Liu JJ, Li H, Li LQ (2018). Contrast-enhanced ultrasound to monitor early recurrence of primary hepatocellular carcinoma after curative treatment. Biomed Res Int..

[4] Forner A, Llovet JM, Bruix J (2012). Hepatocellular carcinoma. Lancet (London England)..

[5] Bruix J, Sherman M (2011). Management of hepatocellular carcinoma: an update. Hepatol(Baltimore Md)..

[6] Yamashita Y, Tsuijita E, Takeishi K, Ishida T, Ikegami T, Ezaki T, Maeda T, Utsunomiya T, Nagasue N, Shirabe K (2014). Trends in surgical results of hepatic resection for hepatocellular carcinoma: 1,000 consecutive cases over 20 years in a single institution. Am J Surg..

[7] Viganò L, Laurenzi A, Solbiati L, Procopio F, Cherqui D, Torzilli G (2018). Open liver resection, laparoscopic liver resection, and percutaneous thermal ablation for patients with solitary small hepatocellular carcinoma (≤30 mm): review of the literature and proposal for a therapeutic strategy. Dig Surg..

[8] Berardi G, Muttillo E, Colasanti M, Mariano G, Meniconi R, Ferretti S, Guglielmo N, Angrisani M, Lucarini A, Garofalo E (2023). Challenging Scenarios and Debated Indications for Laparoscopic Liver Resections for Hepatocellular Carcinoma. Cancers..

[9] Heimbach JK, Kulik LM, Finn RS, Sirlin CB, Abecassis MM, Roberts LR, Zhu AX, Murad MH, Marrero JA (2018). AASLD guidelines for the treatment of hepatocellular carcinoma. Hepatol (Baltimore Md)..

[10] EASL Clinical Practice Guidelines (2018). Management of hepatocellular carcinoma. J Hepatol..

[11] Forner A, Reig ME, de Lope CR, Bruix J (2010). Current strategy for staging and treatment: the BCLC update and future prospects. Semin Liver Dis..

[12] Zhou Y, Zhao Y, Li B, Xu D, Yin Z, Xie F, Yang J (2010). Meta-analysis of radiofrequency ablation versus hepatic resection for small hepatocellular carcinoma. BMC Gastroenterol..

[13] Zhou DC, Geng XP, Zhu LX, Zhao HC, Liu FB, Zhao YJ (2011). Percutaneous radiofrequency ablation versus hepatic resection for small hepatocellular carcinoma: a meta analysis. Zhonghua wai ke za zhi Chin j surg..

[14] Qi X, Tang Y, An D, Bai M, Shi X, Wang J, Han G, Fan D (2014). Radiofrequency ablation versus hepatic resection for small hepatocellular carcinoma: a meta-analysis of randomized controlled trials. J Clin Gastroenterol..

[15] Si MB, Yan PJ, Hao XY, Du ZY, Tian HW, Yang J, Han CW, Yang KH, Guo TK (2019). Efficacy and safety of radiofrequency ablation versus minimally invasive liver surgery for small hepatocellular carcinoma: a systematic review and meta-analysis. Surg Endosc..

[16] Stroup DF, Berlin JA, Morton SC, Olkin I, Williamson GD, Rennie D, Moher D, Becker BJ, Sipe TA, Thacker SB: Meta-analysis of observational studies in epidemiology: a proposal for reporting. Meta-analysis Of Observational Studies in Epidemiology (MOOSE) group Jama 2000, 283(15):2008–2012. 10.1001/jama.283.15.2008 10789670

[17] Moher D, Liberati A, Tetzlaff J, Altman DG (2009). Preferred reporting items for systematic reviews and meta-analyses: the PRISMA statement. BMJ (Clin res ed)..

[18] Llovet JM, Brú C, Bruix J (1999). Prognosis of hepatocellular carcinoma: the BCLC staging classification. Semin Liver Dis..

[19] Tierney JF, Stewart LA, Ghersi D, Burdett S, Sydes MR (2007). Practical methods for incorporating summary time-to-event data into meta-analysis. Trials..

[20] Lee DH, Kim JW, Lee JM, Kim JM, Lee MW, Rhim H, Hur YH, Suh KS (2021). Laparoscopic liver resection versus percutaneous radiofrequency ablation for small single nodular hepatocellular carcinoma: comparison of treatment outcomes. Liver Cancer..

[21] Lai C, Jin RA, Liang X, Cai XJ (2016). Comparison of laparoscopic hepatectomy, percutaneous radiofrequency ablation and open hepatectomy in the treatment of small hepatocellular carcinoma. J Zhejiang Univ Sci B..

[22] Zhang YH, Su B, Sun P, Li RM, Peng XC, Cai J (2020). Percutaneous radiofrequency ablation is superior to hepatic resection in patients with small hepatocellular carcinoma. World J Clin Cases..

[23] Pan YX, Long Q, Yi MJ, Chen JB, Chen JC, Zhang YJ, Xu L, Chen MS, Zhou ZG (2020). Radiofrequency ablation versus laparoscopic hepatectomy for hepatocellular carcinoma: a real world single center study. Eur J Surg Oncol..

[24] Ogiso S, Seo S, Eso Y, Yoh T, Kawai T, Okumura S, Ishii T, Fukumitsu K, Taura K, Seno H (2021). Laparoscopic liver resection versus percutaneous radiofrequency ablation for small hepatocellular carcinoma. HPB (Oxford)..

[25] Di Sandro S, Benuzzi L, Lauterio A, Botta F, De Carlis R, Najjar M, Centonze L, Danieli M, Pezzoli I, Rampoldi A (2019). Single hepatocellular carcinoma approached by curative-intent treatment: a propensity score analysis comparing radiofrequency ablation and liver resection. Eur J Surg Oncol..

[26] Cheng KC, Ho KM (2022). Pure laparoscopic liver resection versus percutaneous radiofrequency ablation for small hepatocellular carcinoma: a propensity score and multivariate analysis. Transl Cancer Res..

[27] Song J, Wang Y, Ma K, Zheng S, Bie P, Xia F, Li X, Li J, Wang X, Chen J (2016). Laparoscopic hepatectomy versus radiofrequency ablation for minimally invasive treatment of single, small hepatocellular carcinomas. Surg Endosc..

[28] Mazzaferro V, Regalia E, Doci R, Andreola S, Pulvirenti A, Bozzetti F, Montalto F, Ammatuna M, Morabito A, Gennari L (1996). Liver transplantation for the treatment of small hepatocellular carcinomas in patients with cirrhosis. N Engl J Med..

[29] Freeman RB, Edwards EB, Harper AM (2006). Waiting list removal rates among patients with chronic and malignant liver diseases. Am J Transplant Off J Am Soc Transplant Am Soc Transplant Surg..

[30] Li X, Wu YS, Chen D, Lin H (2019). Laparoscopic hepatectomy versus radiofrequency ablation for hepatocellular carcinoma: a systematic review and meta-analysis. Cancer Manag Res..

[31] Hu L, Yao L, Li X, Jin P, Yang K, Guo T (2018). Effectiveness and safety of robotic-assisted versus laparoscopic hepatectomy for liver neoplasms: a meta-analysis of retrospective studies. Asian j surg..

[32] Tsukamoto M, Imai K, Yamashita YI, Kitano Y, Okabe H, Nakagawa S, Nitta H, Chikamoto A, Ishiko T, Baba H (2020). Endoscopic hepatic resection and endoscopic radiofrequency ablation as initial treatments for hepatocellular carcinoma within the Milan criteria. Surg Today..

[33] Imai K, Beppu T, Chikamoto A, Mima K, Okabe H, Hayashi H, Nitta H, Ishiko T, Baba H (2014). Salvage treatment for local recurrence of hepatocellular carcinoma after local ablation therapy. Hepatol Res..

[34] Taura K, Ikai I, Hatano E, Fujii H, Uyama N, Shimahara Y (2006). Implication of frequent local ablation therapy for intrahepatic recurrence in prolonged survival of patients with hepatocellular carcinoma undergoing hepatic resection: an analysis of 610 patients over 16 years old. Ann Surg..

[35] Rhim H, Lim HK (2010). Radiofrequency ablation of hepatocellular carcinoma: pros and cons. Gut and liver..

[36] Xu Q, Kobayashi S, Ye X, Meng X (2014). Comparison of hepatic resection and radiofrequency ablation for small hepatocellular carcinoma: a meta-analysis of 16,103 patients. Sci Rep..

[37] NP XR, Woodall CE, Scoggins CR, KM MM, Martin RC (2009). Radiofrequency ablation vs. resection for hepatic colorectal metastasis: therapeutically equivalent?. J Gastrointest Surg..

[38] Fan L, Wang Y, Wu M, Wu T, Deng L, Wang Y, Liu L, An TJ (2023). Laparoscopic common bile duct exploration with primary closure could be safely performed among elderly patients with choledocholithiasis. BMC Geriatr..

[39] Fu XT, Tang Z, Chen JF, Shi YH, Liu WR, Gao Q, Ding GY, Song K, Wang XY, Zhou J (2021). Laparoscopic hepatectomy enhances recovery for small hepatocellular carcinoma with liver cirrhosis by postoperative inflammatory response attenuation: a propensity score matching analysis with a conventional open approach. Surg Endosc..

[40] Farges O, Malassagne B, Flejou JF, Balzan S, Sauvanet A, Belghiti J (1999). Risk of major liver resection in patients with underlying chronic liver disease: a reappraisal. Ann Surg..

[41] Kanazawa A, Tsukamoto T, Shimizu S, Kodai S, Yamazoe S, Yamamoto S, Kubo S (2013). Impact of laparoscopic liver resection for hepatocellular carcinoma with F4-liver cirrhosis. Surg Endosc..

[42] Hackl C, Schlitt HJ, Renner P, Lang SA (2016). Liver surgery in cirrhosis and portal hypertension. World J Gastroenterol..

[43] Zhu AX (2012). Molecularly targeted therapy for advanced hepatocellular carcinoma in 2012: current status and future perspectives. Semin Oncol..

