# Supplementary material for: Comparison of laparoscopic hepatectomy and percutaneous radiofrequency ablation for the treatment of small hepatocellular carcinoma: a meta-analysis
Source: BMC Surg. 2024 Mar 5;24:83. doi: 10.1186/s12893-024-02376-5 (PMC10913421; doi:10.1186/s12893-024-02376-5)
Supplement: Supplementary file 1 — Supplementary Material 1. [file 12893_2024_2376_MOESM1_ESM.pptx]

## Slide 1
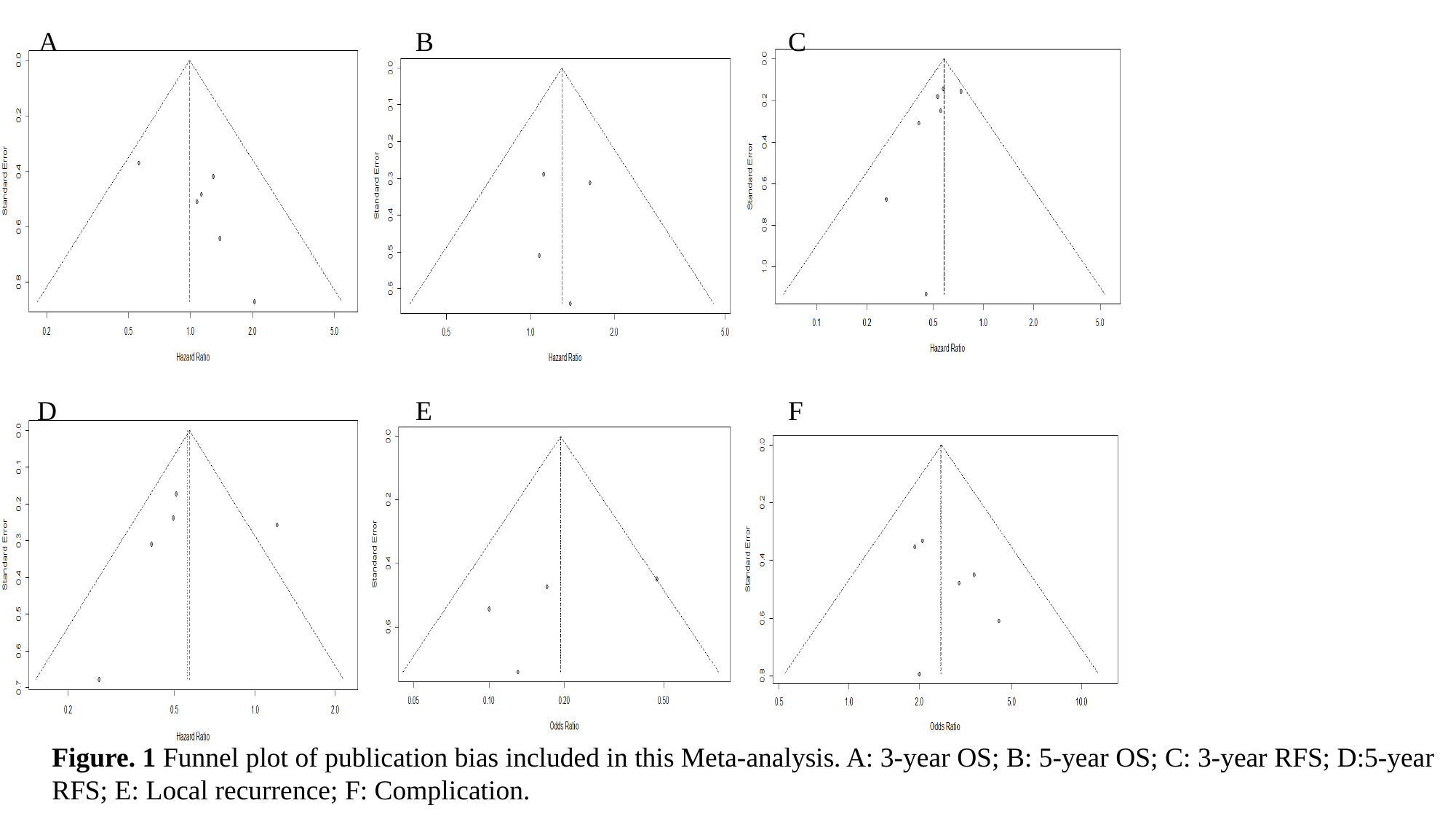

A
B
C
D
E
F
Figure. 1 Funnel plot of publication bias included in this Meta-analysis. A: 3-year OS; B: 5-year OS; C: 3-year RFS; D:5-year RFS; E: Local recurrence; F: Complication.
